# The RNA-binding protein Modulo promotes neural stem cell maintenance in *Drosophila*

**DOI:** 10.1371/journal.pone.0309221

**Published:** 2024-12-19

**Authors:** Amalia S. Parra, Christopher A. Johnston

**Affiliations:** 1 Department of Biology, U.S Department of Energy, (DOE), Oakridge Institute for Science and Education, (ORISE), Office of the Director of National Intelligence, (ODNI), University of New Mexico, Albuquerque, New Mexico, United States of America; 2 Department of Biology, University of New Mexico, Albuquerque, New Mexico, United States of America; University of California Santa Barbara, UNITED STATES OF AMERICA

## Abstract

A small population of stem cells in the developing *Drosophila* central nervous system generates the large number of different cell types that make up the adult brain. To achieve this, these neural stem cells (neuroblasts, NBs) divide asymmetrically to produce non-identical daughter cells. The balance between stem cell self-renewal and neural differentiation is regulated by various cellular machinery, including transcription factors, chromatin remodelers, and RNA-binding proteins. The list of these components remains incomplete, and the mechanisms regulating their function are not fully understood, however. Here, we identify a role for the RNA-binding protein Modulo (Mod; nucleolin in humans) in NB maintenance. We employ transcriptomic analyses to identify RNA targets of Mod and assess changes in global gene expression following its knockdown, results of which suggest a link with notable proneural genes and those essential for neurogenesis. Mod is expressed in larval brains and its loss leads to a significant decrease in the number of central brain NBs. Stem cells that remain lack expression of key NB identity factors and exhibit cell proliferation defects. Mechanistically, our analysis suggests these deficiencies arise at least in part from altered cell cycle progression, with a proportion of NBs arresting prior to mitosis. Overall, our data show that Mod function is essential for neural stem cell maintenance during neurogenesis.

## Introduction

**Fig 1:**
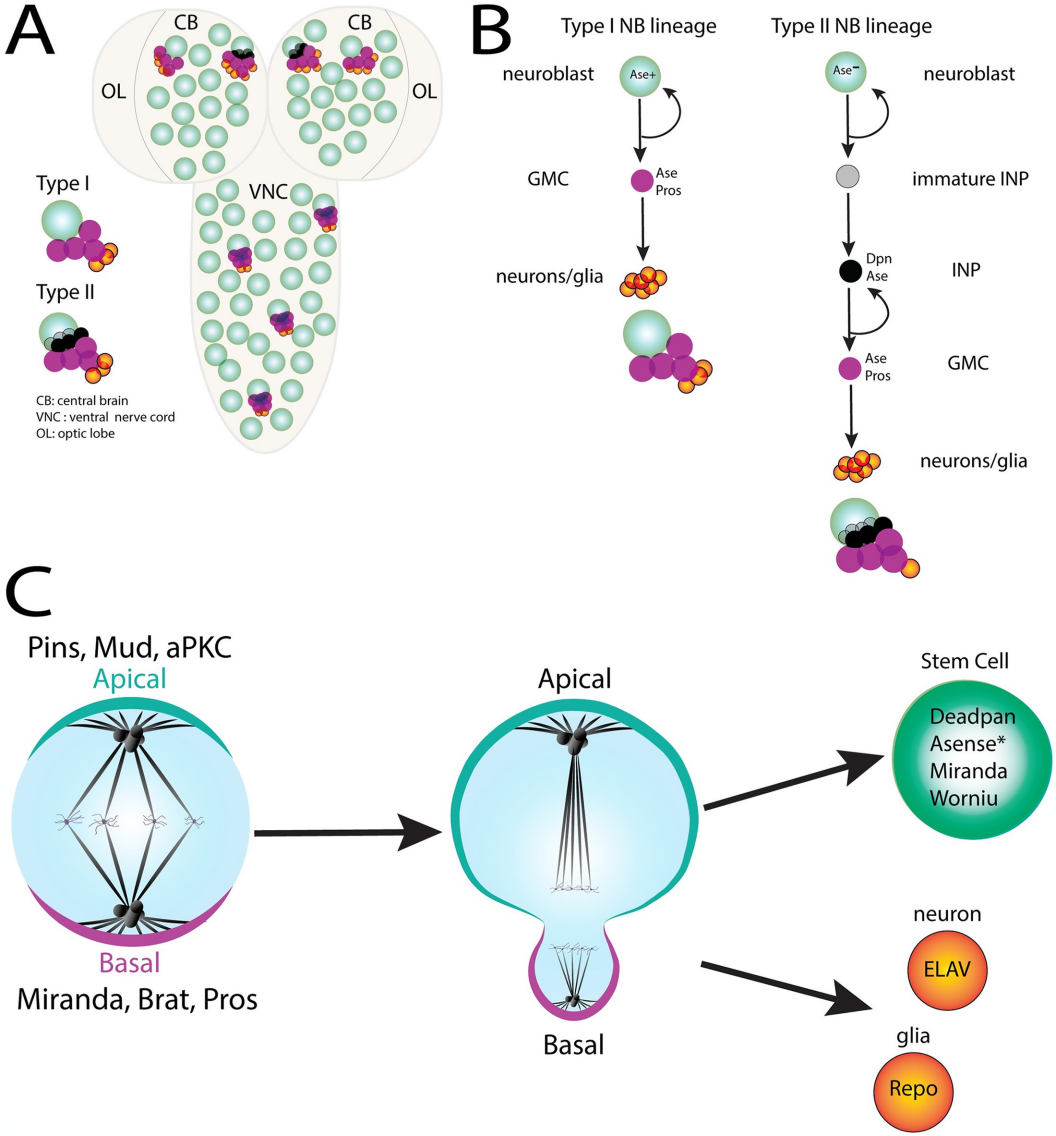
Development of *Drosophila* Neuroblasts (NB). ** (A)** Schematic of the *Drosophila* larval central nervous system (CNS) consisting of the ventral nerve cord (VNC) and two brain lobes. Each lobe contains a central brain (CB) region and an optic lobe (OL) region. Neuroblast (NB) populations in these regions consist of Type I and Type II NB lineages. **(B)** Lineage map of Type I and Type II larval NBs. The Type I lineage undergoes self-renewal and subsequent differentiation directly via ganglion mother cells (GMC), which divide to produce neurons or glia. Type II NBs also self-renew; however, they generate an intermediate neural progenitor (INP) cell that itself undergoes self-renewing divisions to produce the GMC. Both lineages produce glia and neurons for the adult fly CNS. **(C)** Schematic depicting asymmetric cell division (ACD) in NBs. This process relies on establishment of cell polarity and positioning of the mitotic spindle along the polarity axis to promote asymmetric segregation of cell fate determinants (i.e apical: Pins, aPKC, Mud; basal: Miranda, Brat, Pros). A self-renewing neuroblast (expressing Deadpan, Asense, Worniu, and Miranda) and a GMC (Type I lineage) or an INP (Type II lineage) result from these divisions, ultimately leading to production of ELAV+ neurons or Repo+ glia.

Regulation of tissue size and architecture, along with fate specification of distinct constituent cell types, is fundamental to ensure proper tissue development. One way this is achieved is through coordination of cell growth and proliferation with cell identity cues. A noteworthy example are the neural stem cells (neuroblasts; NBs) in the *Drosophila melanogaster* central nervous system (CNS). The three main categories of NBs: embryonic, larval central brain, and larval optic lobe, give origin to the specialized cell types found in the CNS. Larval central brain neuroblasts include 90–100 Type I NBs and 8 Type II NBs, which differ in their specific modes of neurogenesis [1]. Proliferation of NBs is highly regulated throughout development. Embryonic NBs divide rapidly prior to entering a dormant state. Developmentally-timed environmental and genetic signals induce a transient quiescence followed by reinitiated proliferation during the embryonic-larval transition. Specifically, NBs remain quiescent for ~24 hours and proliferation resumes in response to signals from a hepatic-like tissue called the fat body to begin larval neurogenesis [2–5]. Here, nutrient-rich conditions lead to NB enlargement and cell cycle re-entry. Re-activation of embryonic NBs marks the onset of larval neurogenesis in which larval NBs develop and ultimately produce adult CNS components [4]. During this time, NBs undergo a series of asymmetric cell divisions that define an extended period of larval neurogenesis [6,7]. Briefly, *Drosophila* NBs divide asymmetrically to produce a larger daughter cell that inherits the Par complex, including atypical protein kinase C (aPKC), that promotes stem cell self-renewal [8–11] (Fig 1). The smaller daughter cell, the ganglion mother cell (GMC), contains the differentiation factors Prospero (Pros), Brat, and Numb and divides to produce neurons and glial cells [12–15]. Aside from these factors, key NB maintenance components such as Deadpan (Dpn), Asense (Ase), and Worniu (Wor) confer NB identity to the larger cell [16,17]. The ability to self-renew is conserved in both Type I and Type II NB lineages. Unlike Type I NBs, however, Type II NBs produce self-renewing intermediate neural progenitor (INP) cells that produce GMCs to generate differentiated progeny (Fig 1B) [18]. Differences in transcriptional profiles also define the two lineages. For instance, the Type I lineage expresses the NB factors Ase and Dpn, while the Type II lineage expresses Dpn but not Ase and gives rise to progeny that are Dpn+ Ase+ Pros+ (e.g. INPs). These differences in gene expression assure that proliferation potential and stem cell properties are conferred on the correct cells. Dysfunction of cell fate determinants or loss of NB identity factors can result in depletion of the stem cell pool through various mechanisms including loss of stemness, premature differentiation, and prolonged quiescence [19–21]. Conversely, aberrant expression of pro-growth and proliferative genes can lead to excessive growth of some tissues [22,23]. Together, these mechanisms point to the importance of spatial and temporal coordination of cell fate determinants and cell proliferation cues to achieve proper tissue development and architecture.

As a highly active transcriptional center, the nucleolus has an important role in coordinating multiple processes in the cell, including stem cell proliferation, cell fate determination, stress responses, and mitotic progression [24]. In prophase, nuclear proteins released during nucleolar disassembly aid in cell cycle progression and institution of appropriate stem cell profiles [25,26]. Expression of transcriptional repertoires that are consistent with cell type and developmental stage are pivotal in formation of complex tissues. For instance, highly proliferative cells such as embryonic stem cells (ESCs) initially exhibit high levels of rRNA transcription and large nuclei, but convert to lower levels of transcription and numerous, smaller, nuclei upon differentiation [27]. This conversion engages the large nucleolar proteome (>1300 identified in humans, [28]) that is made up of RNA-binding proteins, translation initiation factors, elongation factors, and chromatin remodelers. These components are also responsible for other nucleolar functions including ribosome biogenesis, nuclear stress responses, and genome organization [29–32].

One of the key proteins released into the cytoplasm during nucleolar breakdown is nucleolin. In humans, nucleolin promotes stem cell self-renewal and proliferation by regulating cell cycle progression and preventing differentiation in ESCs [33]. Similarly, in fruit flies, the nucleolar proteins Nucleostemin and Brain tumor (brat) regulate stem cell proliferation and homeostasis of ESCs and adult stem cells [34–36]. These regulatory tasks are thought to occur via nucleolar sequestration of genes and critical factors during interphase and morphological changes throughout development and across cell types [37,38]. Collectively, these studies demonstrate that stem cell homeostasis is regulated by many of the known nucleolar processes, including protein synthesis, cell cycle progression, and ribosome biogenesis. These nucleolar functions influence the translational program of stem cells to control their function and maintenance. Specifics of how the nucleolus performs these functions requires further analysis of key nucleolar proteins.

**Fig 2:**
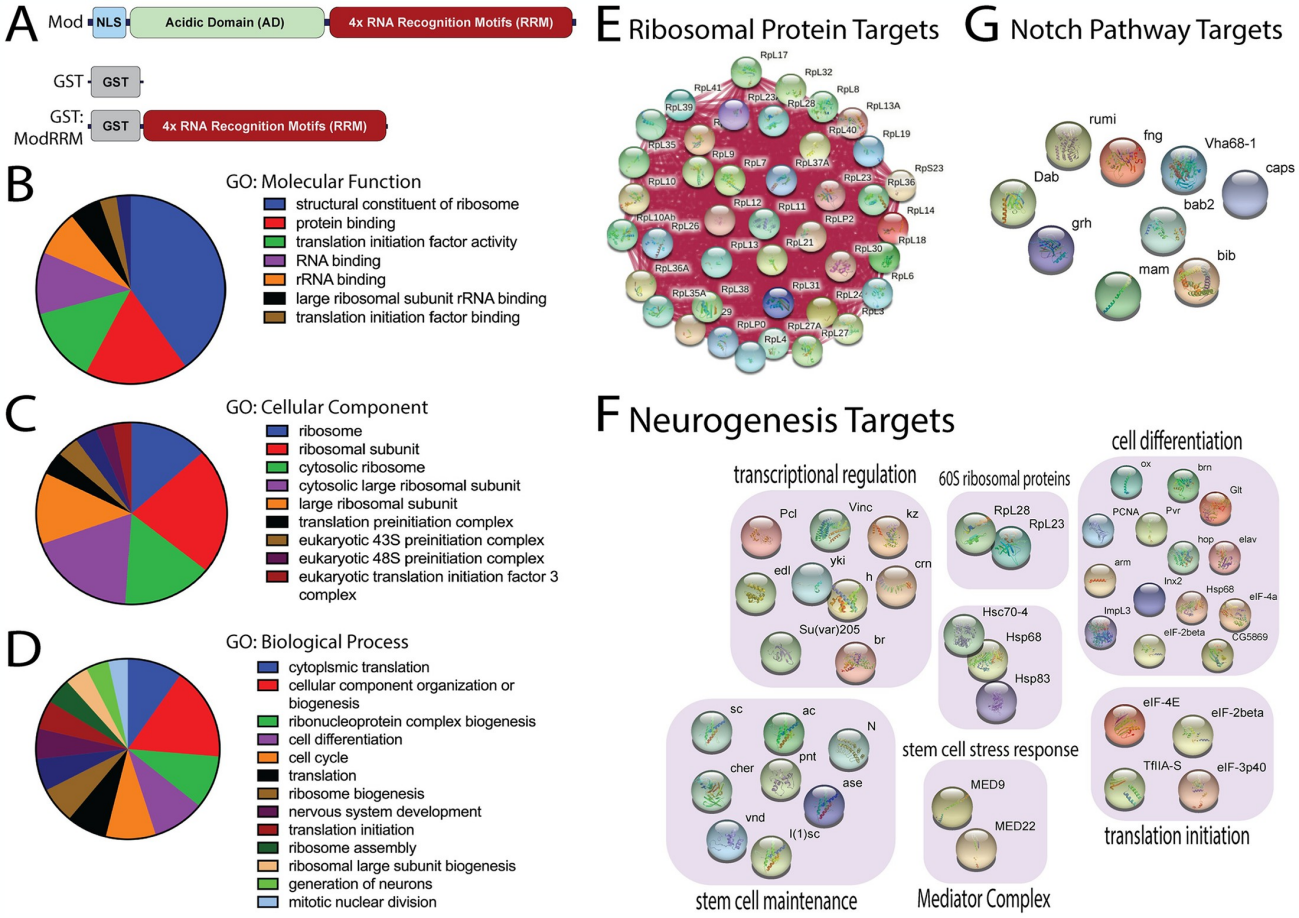
RNA targets and biological process GO terms associated with Mod RiP-Seq analysis. **(A)** Domain architecture of Mod full length protein showing a nuclear localization signal (NLS), the disordered, low-complexity acidic domains (AD), and four RNA recognition motifs (RRM). Domain diagrams of GST (negative control) and GST-ModRRM recombinant proteins used in RiP-Seq target identification analysis are also depicted. **(B-D)** Mod targets were identified using RNA immunoprecipitation coupled to Sequencing (RiP-Seq), and DAVID Bioinformatic 6.8 was used to assign indicated Gene Ontology (GO) terms. Wedge sizes correspond to significance (*p*<0.001-*p*<0.05), and each wedge represents at least eight genes. Panels represent GO terms for Molecular function **(B)**, Cellular Component **(C)**, and Biological Process **(D)**. **(E)** Select Ribosomal protein targets of Mod are shown along with their network of molecular interactions. **(F)** Select targets of Mod involved in indicated cellular processes essential for neurogenesis. **(G)** Notch pathway genes identified as Mod targets.

The nucleolar protein Modulo (Mod) is the fly ortholog of the well-studied nucleolin gene in humans. Mod was initially identified as a DNA-binding protein with varying expression throughout *Drosophila* embryogenesis. It was later characterized as a dominant suppressor of position effect variegation (PEV) and an RNA-binding protein [39,40]. Mod contains four RNA recognition motifs (RRM) believed to bind RNA and an N-terminal nuclear localization signal (NLS) adjacent to an acidic domain (AD) (Fig 2A). Mod binds DNA/protein complexes involved in chromatin remodeling and may play a role in gene expression [40]. Additionally, Mod is required for proper morphogenesis in early embryonic development and regulation of pattern forming genes [41]. Apart from its role in chromatin modification, Mod also regulates nucleolar activity and thus is required for proper growth and regulation of proliferative tissues [42,43]. To date, the role of Mod/Nucleolin in development has been frequently linked to its interaction with Myc and regulation of cell proliferation [44]. A specific mechanism for Mod-mediated regulation of cell growth, including in stem cells, however, has not been determined, nor has any extensive analysis of its RNA targets been undertaken. It is important to note that RNA binding proteins have emerged as an important family of proteins controlling stem cell function [45]. Furthermore, several of these proteins have now been established as important regulators of neural stem cell function in *Drosophila* [46–48]. Thus, while Mod may play a multifaceted role, it is particularly important to evaluate the role of its principal RNA binding function.

Here we perform transcriptomic analyses to identify RNA targets of Mod and genome-wide changes in gene expression following Mod knockdown. In addition to predicted ribosomal and cell growth-related genes, we show that Mod unexpectedly binds well-established proneural gene clusters along with numerous genes involved in neurogenesis, including those promoting stem cell identity. We also describe a role for Mod in *Drosophila* NB homeostasis, with loss of Mod leading to a reduction in the NB pool. This loss is characterized further by transcriptional changes incompatible with maintenance of NB identity and alterations in cell cycle progression. Overall, our results suggest Mod is required for maintenance of central brain NBs, which may act in part through regulation of essential neural stem cell identity genes.

## Methods

### *Drosophila melanogaster* husbandry and genetics

*Drosophila melanogaster* stocks were entrained to 12-hr light-12-12-hr dark cycles, maintained at a temperature of 20°C and relative humidity of 45–50%, and routinely checked for mite contamination. Crosses were raised at 29°C under similar conditions for all experiments unless otherwise noted. For developmental timing experiments, embryos were collected on yeasted grape juice plates and allowed to develop to the appropriate larval stage. Larvae were then transferred to a food plate (t = 0 hours ALH) and allowed to develop to the desired stage.

### Antibody staining

Whole brains from third instar larvae were dissected in cold (4°C) PBS followed by fixation for 25 minutes in 4% paraformaldehyde at room temperature (RT). Tissues were washed three times for 10 minutes in PBS-T (1x PBS, 0.3% Triton) and blocked for 1 hour at RT (1x PBS, 0.3% Triton, 2.5% goat serum, 2.5% donkey serum) then incubated overnight in primary antibody solution at 4°C. Following incubation, tissues were washed three times for 20 minutes in PBS supplemented with donkey and goat serum, followed by incubation in secondary antibody for 2 hours at RT. Following incubation, larval brains were washed three times for ten minutes in PBS-T then mounted ventral side up in 80% glycerol and stored at 4°C until imaging.

The following antibodies were used: Guinea pig anti-Deadpan (1:1000) (generous gift from J. Skeath), Rat anti-Deadpan (1:100) (Abcam, #195173), Rat Anti-Miranda (1:500) (Abcam, #197788),Rabbit Anti-Fibrillarin (1:1000) (Abcam, #5821), Mouse anti-ELAV-9F8A9 (1:50) (DSHB), Mouse 8D12 Anti-Repo (1:50) (DSHB), Mouse Anti-Lamin (1:50) (DSHB,ADL67.10), Rabbit Anti-PH3 (1:1000) (Invitrogen, #PA5-17869), Rabbit Anti-PKC (1:1000) (Santa Cruz Biotechnology, #sc216), Rabbit Anti-Asense (1:400; generous gift from C.Y. Lee, University of Michigan), and Rabbit Anti-Mod (custom produced by YenZym, San Francisco, CA, this study).

### Fly stocks

The *1407inscuteableGAL4* was used as a driver line throughout the study (BDSC, #8751). An additional double transgenic line *1407inscuteableGAL4/1407inscuteableGAL4;UAS-modRNAi/UAS-modRNAi* was created in this study using a *cyo/Br;TM2/TM6* double balancer line (generous gift from R.M. Cripps, San Diego State University) and used where indicated. Two independent UAS-*modRNAi* lines were used (BDSC, #28314 and VDRC, #330594), as well as the UAS-p35 (BDSC, #5072) as transgenic lines crossed to the 1407 driver. The *modL8* allele (BDSC, #38432) was used as an additional loss-of-function line. Wildtype *yw* stock, used for Control crosses, was a generous gift (C.Q. Doe, University of Oregon).

### Cell lines

*Drosophila* Schneider 2 (S2) cells (Invitrogen) were maintained at 27°C in Schneider Insect Medium (SIM; Invitrogen) supplemented with 10% fetal bovine serum and passaged every 3–4 days. Cells were routinely monitored for mycoplasma contamination.

### RNAi of *Drosophila* S2 cells

RNAi primers containing T7 promoter sequence recognition tags were designed to amplify segments of ~200–600 base pairs using SnapDragon (http://www.flyrnai.org/snapdragon). Target sequences were PCR-amplified to yield double-stranded RNA using the MEGAscript T7 kit (ThermoFisher, cat#AM1333) and accompanying protocol. Amplified segments were designed to recognize all isoforms of the target transcripts.

S2 cells were seeded in six-well dishes at 1 x106 cells per well with 1mL of serum-free SIM and treated with 40μg of dsRNA targeted against *modulo*. Cells were incubated at 27°C for 1 h followed by addition of 2mL of serum-containing SIM. Cells were incubated at 27°C for 3–5 days prior to downstream applications.

### Recombinant protein expression

Coding sequence for the ModRRM domains was PCR amplified with BamHI/XhoI restriction sites using an S2 cell cDNA library template. Following enzyme digestion, products were ligated into pGEX backbone to generate a GST-ModRRM fusion that was sequenced confirmed using standard methods (McLab Laboratories, San Francisco, CA). Plasmid was transformed into BL21(DE3) competent *E*. *coli* cells (ThermoFisher, #C600003) followed by culturing at 37°C in LB supplemented with 100μg /mL ampicillin. Cultures were grown to an OD600 of ~0.6 and induced with 0.2mM Isopropyl ß-D-1-thiogalactopyranoside and grown overnight at 18°C. Bacterial pellets were resuspended in cold PBS and lysates were prepared using a sonicator (Branson Digital, Danbury Connecticut).

### RNA-Immunoprecipitation and RNA-Sequencing (RiP-Seq) analysis

GST-ModRRM or GST alone were coupled to glutathione agarose for 1h at room temperature followed by extensive washing with PBS-T (1x PBS, 0.2% Triton). S2 cells were collected via centrifugation at 1000g for 3 minutes followed by washing three times with cold PBS. Cells were resuspended in buffer and lysed using 25 strokes of a Dounce homogenizer followed by centrifugation at 4000g for 30 minutes [49]. Resulting supernatant was used for sucrose density ultracentrifugation as described. Protein-RNA complex was prepared by combining equal volumes of supernatant above the S130-interace with GST alone or GST-ModRRM at 4°C for 1 hour. Protein-RNA complex was eluted from the agarose beads with 1% SDS and boiling for 3 minutes.

RNA was extracted using Phenol/Chloroform along with the RNeasy Midi Kit (Qiagen, #75144) followed by digestion with RNase-free DNase. RNA sequencing was performed on two biological replicates using the Illumina Next Generation Sequencing platform (Illumina). Libraries were prepared using 500ng total RNA and a KAPA mRNA Hyper Prep kit (Roche). Raw reads were trimmed using Trimmomatic v0.36 and high-quality reads were mapped to the *Drosophila melanogaster* genome using STAR. Transcript expression was analyzed using featureCounts and genes with an adjusted *p* ≤ 0.05 with an FPKM >2 were considered for further analysis.

### Differential gene expression analyses using RNA-Sequencing

RNA was extracted as described above with the following modifications. 500ng total RNA was used along with a RNeasy Mini Kit (Qiagen). Raw reads were trimmed and filtered using Trimmomatic v0.36 (Bolger et. al. 2014) with slide window of 4 nt, average score above 20 and minimum length of 36 nt. High quality reads were mapped to the *D*. *melanogaster* genome (NCBI version GCA_000001215.4 Release 6 plus ISO1 MT) using STAR v2.5.3a (Dobin et.al 2013). Transcript expression levels were estimated using featureCounts v1.6.2 and differential gene expression analysis was performed using EBSeq v1.18.0 (Leng et. al 2013). Genes with an adjusted *p* ≤ 0.05 with and log2fold change >1 (upregulated) or <1 (downregulated) were considered for further analysis.

### Image acquisition and processing

Images were acquired using a Zeiss LSM-780 confocal microscope utilizing a 40x/0.65 NA oil-immersion objective. All images were processed using Fiji and Adobe Photoshop software and figures were assembled in Adobe Illustrator.

NB diameters were quantified as previously described [50,51]. Briefly, individual confocal image slices from z-projections were used to measure two perpendicular diameters through the center of the NB. The final diameters are reported as the average of these two independent measurements. These diameters were acquired using the Line Tool and Measure functions in Fiji software. NB number counts and diameter measurements were verified by a second experimenter using coded images.

All data reported are from at least 5 independent replicates and the statistical methods used are indicated in respective figure legends.

## Quantification and statistical analysis

### Statistical analysis

All statistical analyses were performed using Graph Pad Software (v 9.0).

## Results and discussion

### Identification of Mod targets reveals genes involved in cell growth and neurogenesis

Mod is a conserved nucleolar RNA-binding protein expressed throughout *Drosophila* development and has roles in germ cell differentiation and cell growth and proliferation of epithelia [44,52]. In mammalian tissues, Nucleolin is involved in rRNA synthesis, stem cell self-renewal during early development [33,53,54], regulation of mRNA localization in axons, and hematopoietic stem cell maintenance [55]. Despite these studies highlighting the importance of Mod/Nucleolin in diverse cellular processes, a comprehensive analysis of their RNA targets has not been performed. Molecular details of how Mod functions to promote cell differentiation and tissue growth could potentially be revealed by identifying its RNA targets. To do this, we performed RNA-Immunoprecipitation coupled to Sequencing (RiP-Seq) in *Drosophila* Schneider 2 (S2) cells, which are known to express nucleolar-localized Mod [56]. These phagocytic cells are derived from late-stage *Drosophila* embryos and express many of the annotated *Drosophila* genes, making them ideal for immunoprecipitation studies and an unbiased elucidation of biochemical processes [57,58]. We incubated a recombinant GST-tagged Mod protein spanning the four tandem RRM domains (Mod-RRM) or GST alone (Fig 2A) control protein with RNA isolated from total cell lysate to allow protein/RNA complex formation, followed by RNA purification and sequencing (S1 Table; see Methods for details).

As anticipated, analysis revealed genes enriched for Gene Ontology (GO) terms of ribosome biogenesis and protein synthesis, similar to functions associated with its human homologue nucleolin [59]. Other highly enriched targets included those belonging to ribosomal RNAs (rRNA), microRNA (miRNAs), small nucleolar RNAs (snoRNA), and small Cajal body-specific RNA (scaRNA), target categories that are also conserved in nucleolin (Fig 2B–2D and S1 Table) [60,61]. Specific targets of interest revealed in this analysis include those involved in regulating nucleolar dynamics and ribosome functions, such as stubarista (sta), MYB binding protein 1a (Mybbp1A), and eukaryotic translation elongation factor 1 alpha 1 (eEF1α1). A complete list of Mod target RNAs is provided in S1 Table. Unexpectedly, our target analysis also revealed novel GO categories not previously known for Mod or nucleolin, including neurogenesis and regulation of cell identity (Fig 2F). Core cell identity factors such as Notch (N), achaete (ac), scute (sc), Ase, and members of the Mediator Complex were among genes detected having a previously detailed link to neural stem cell function (Fig 2F and 2G) [62]. Keeping with the role in cell growth, our analysis also revealed important regulators of cell growth such as RhoGAP1A, Retinoblastoma-family protein (Rbf), SKP1-related A (SkpA), and Mod itself [63]. More broadly, factors involved in early stem cell development were also identified, including the H3K9 methyltransferase G9A, amyloid precursor -like (Appl), frizzled 3 (fz3), and ventral nervous system defective (vnd). Targets necessary for nuclear transport such as ellipsoid body open (ebo) were also highly enriched. Mod also bound transcripts of genes involved in fundamental cellular processes including metabolic enzyme function (COX1, COX3, CYTB) and peptide synthesis (svr, eIF4E7, RpL41). Having linked Mod to several important cellular functions through this target analysis, we proceeded to analyze gene expression profiles in Mod-depleted cells to better understand the potential impact of Mod on processes associated with the targets identified here.

### Loss of Mod leads to transcriptome changes

**Fig 3:**
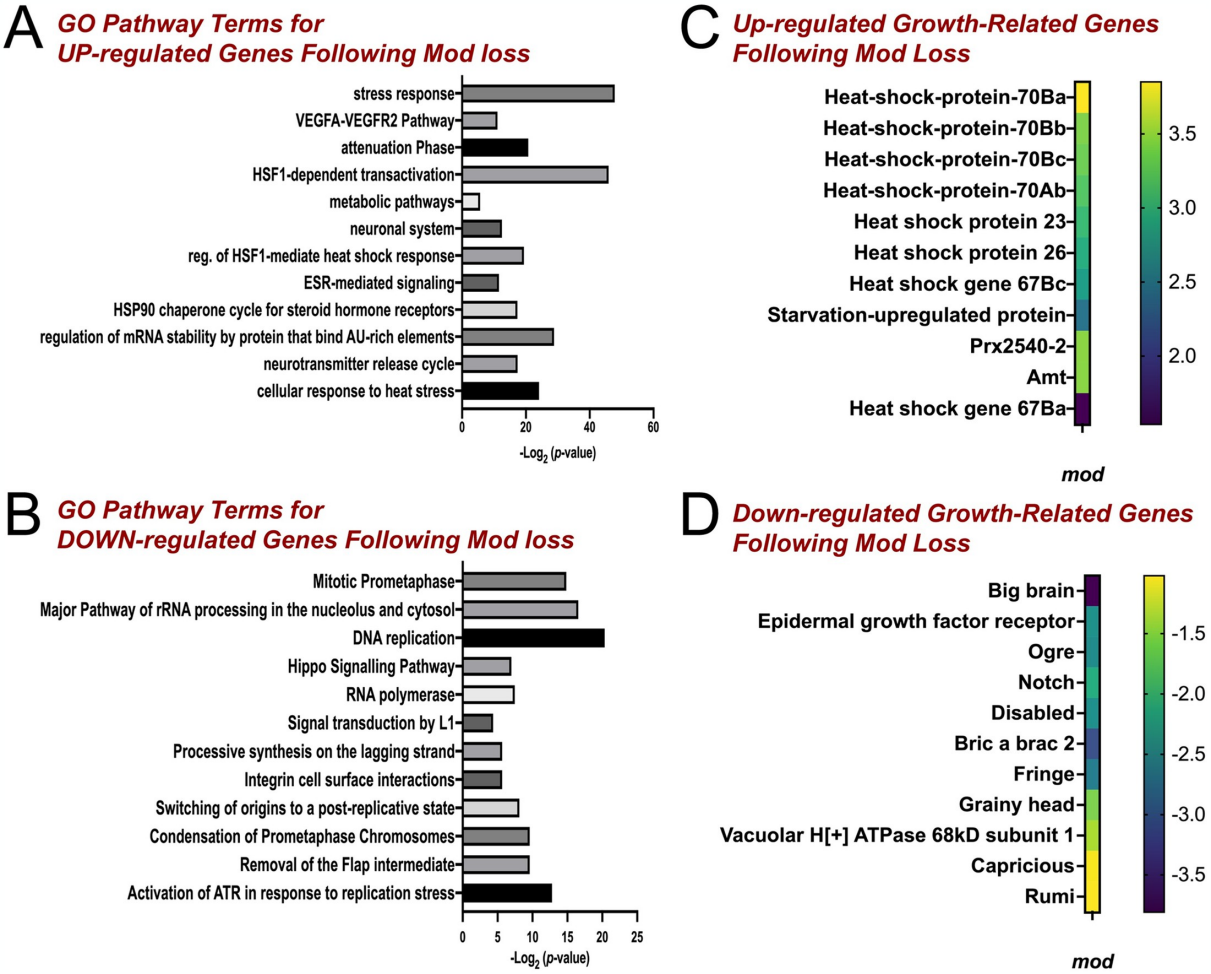
Differential gene expression analysis following Mod knockdown. Plots showing molecular pathways represented by up-regulated genes **(A)** and down-regulated genes **(B)** from DGE analysis of *modRNAi*-treated cells compared to untreated control. **(C)** Select up-regulated genes in Mod-depleted cells relative to untreated control highlights key stress response genes, including those for numerous heat shock proteins. **(D)** Select down-regulated genes in Mod-depleted cells relative to untreated control depicts growth-related genes involved in NB growth and proliferation. Enriched pathway data were generated using DAVID Gene Ontology Analysis with a Bonferroni-adjusted *p*-value ≤ 0.05.

RNA-binding proteins control target activity in numerous ways, which can ultimately influence gene expression [64]. Furthermore, Mod has also been characterized as a DNA- and chromatin-binding protein that could provide additional roles in regulating gene expression [43]. As with its RNA targets, however, little is known about how Mod impacts genome wide expression patterns. To address this, we next performed differential gene expression (DGE) analysis following Mod knockdown in *Drosophila* S2 cells. Total RNA extracted from control cells or those treated with dsRNA against Mod (ModRNAi) was used as input to prepare cDNA libraries for Illumina sequencing. Using a cutoff of log2 > 1, we found 214 upregulated genes and 477 downregulated genes (log2 > -1). A full list of DE genes can be found in S2 Table. Analysis of upregulated genes in Mod-depleted cells revealed an abundance of factors involved in cellular stress responses mediated by heat shock proteins (HSPs; Fig 3A and 3B). These chaperone proteins are produced in response to physical, environmental, and chemical stressors. They cooperate with other cellular machinery to regulate cell growth and promote survival, particularly in neurodevelopment [65]. This is consistent with concomitant upregulation of heat shock factor-1-mediated gene transactivation. Among the HSPs highly upregulated in our analysis were those involved in regulation of protein synthesis under stress and neuroprotection preceding stress, including Hsp23 and Hsp70 (Fig 3B) [66]. The Hsp70 response is also initiated following decreases in protein synthesis resulting from rRNA production defects [67]. Lastly, we found HSPs involved in response to starvation and extending fly life span to be upregulated in Mod-deficient cells [68,69], consistent with a stress response to growth-limiting conditions. Loss of Mod also caused increased expression of PDGF and VEGF-related factor 1 (Pvf1), an activator of Ras/Raf/MAP kinases [70]. Genes involved in specification of neuronal cell types were also observed through increased expression of H6-like-homeobox (Hmx) as well as sanpodo (Spdo) that promote Notch signaling and asymmetric divisions of neural precursor cells [71]. Nervy (nvy), another regulator of N and a suppressor of Ase was also highly expressed [72]. Together, these results suggest that Mod is involved in response to stress, cell fate determination, and cell growth and proliferation.

Pathway analysis of down-regulated genes revealed mechanisms involved in DNA replication and rRNA processing (Fig 3C and 3D). Regarding the downregulation of rRNA processing genes, decreases in rRNA transcription trigger inappropriate stem cell differentiation in mammalian stem cells independent of cell cycle arrest [73]. Transcription of rRNA is essential for ribosome biogenesis and serves not only to maintain nucleolar structure but also to regulate other cell functions like cell cycle progression, protein synthesis, cell proliferation, and stress responses. Interestingly, downregulated genes also included several that are involved not only in cell growth and proliferation but also establishment and maintenance of stem cell identity. Amongst these were key components of the Notch pathway (i.e N, big brain [bib], elbow B [elB], fringe [fng]), which has established roles in maintenance of stem cell identity and formation through its control of self-renewal and differentiation (Fig 3D). We also observed significant downregulation of optic ganglion reduced (Ogre), an essential protein for post-embryonic NB growth and reactivation following quiescence and maverick, a component of BMP signaling [74,75]. These findings are consistent with a role for Mod in rRNA processing and nucleolar homeostasis, both of which align with Mod localization. Along with GO analysis from RiP-seq results described above, downregulation of numerous genes associated with neural stem cell maintenance suggest a potential, unexpected role for Mod in NB function, which we explore further below.

### Mod is expressed throughout the larval CNS

**Fig 4:**
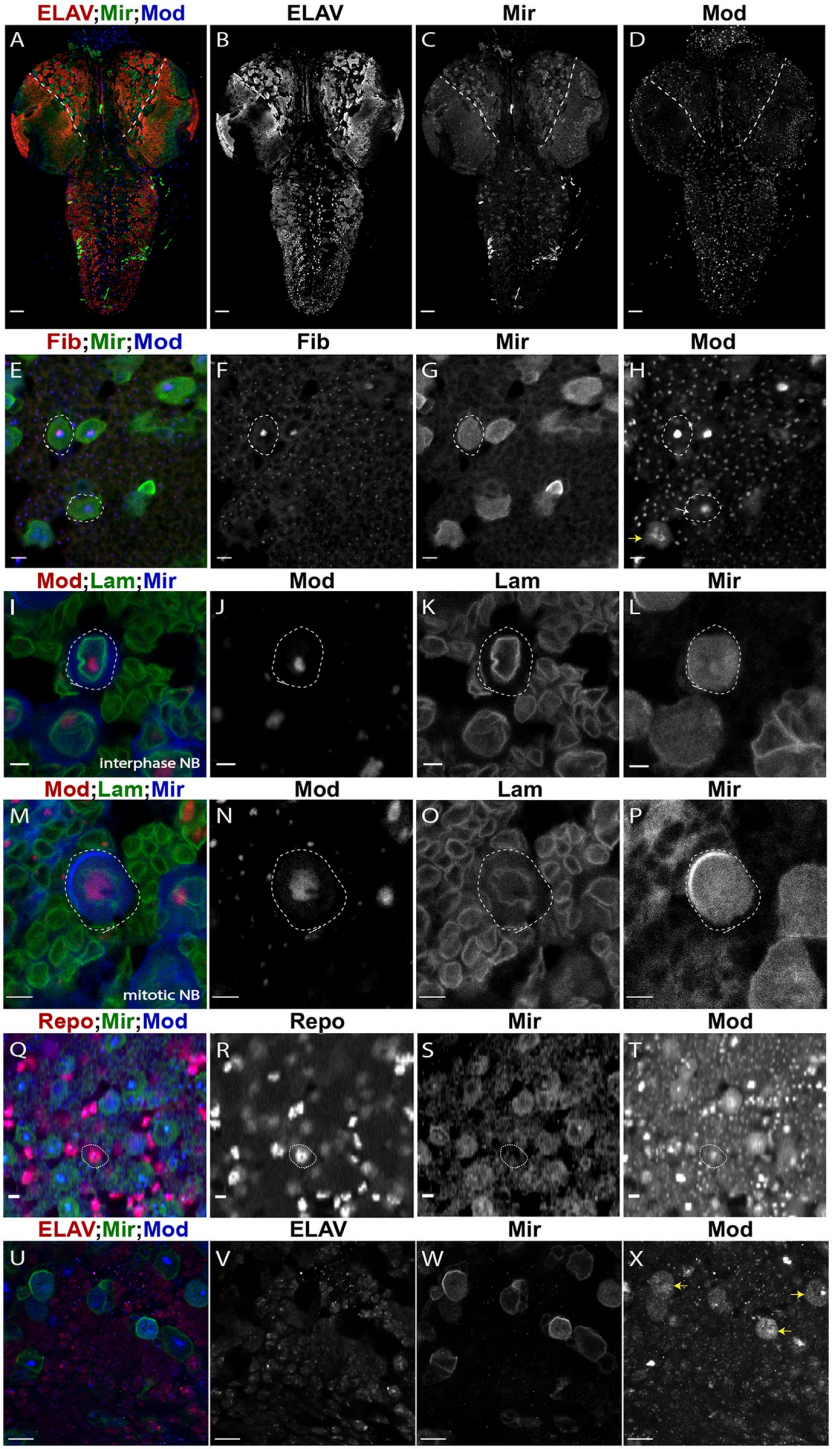
Mod is expressed throughout the larval CNS and is primarily nuclear. ** (A)** Image of fly CNS (Red: ELAV, Green: Mir, Blue: Mod) in Control (1407>*yw*). The central brain (CB) area is medial with respect to dashed lines. Individual channels are depicted in greyscale in **(B-D)**. Scale bars represent 20μm. **(E)** Mod localizes to a subnuclear region consistent with the nucleolus (Red: Fibrillarin (Fib), Green: Mir, Blue: Mod). Individual channels are depicted in greyscale in **(F-H)**. In panel **(H)**, white arrow indicates extra-nucleolar Mod localization, and the yellow arrow indicates a mitotic NB with strong Mod signal at the apparent perichromosomal region as well as throughout the cytoplasm. **(I)** Mod shows prominent localization within a nuclear region in interphase NBs (Red: Mod, Green: Lamin (Lam), Blue: Mir). Individual channels are depicted in gray scale **(J-L)**. **(M)** Mod is localized peri-chromosomally and diffusely cytoplasmic in mitotic NBs (Red: Mod, Green: Lam, Blue: Mir). Individual channels are shown in grayscale **(N-P)**. **(Q)** Mod is expressed in Repo+ glial cells (Red: Repo, Green: Mir, Blue: Mod). Individual channels are represented in greyscale in panels **(R-T)**. Scale bars represent 5μm for panels **(E-T)**. **(U)** Mod is expressed in ELAV+ neuronal cells (Red: ELAV, Green: Mir, Blue: Mod). Individual channels are represented in greyscale in panels **(V-X)**. In panel **(X)**, yellow arrows indicate additional mitotic NBs showing Mod signal at the perichromosomal region as well as throughout the cytoplasm. Scale bars represent 10μm.

Both Mod and nucleolin have been implicated in regulation of cell proliferation in epithelial tissues; however, a thorough analysis of a role in stem cells has not been conducted. Based on our RiP-Seq results, as well as notable gene expression changes following Mod loss, we decided to focus our remaining studies on the role of Mod in *Drosophila* CNS development. Considering the number of critical CNS patterning genes and NB identity factors identified in our RiP-Seq analysis and their importance in NB maintenance, we investigated a role for Mod in larval CNS development, which is characterized by extensive neurogenesis. We first examined Mod expression in the CNS of third instar larvae (L3), a developmental time during which NBs have exited quiescence and undergo extensive proliferation prior to their terminal differentiation [76]. We co-stained brains dissected from wild-type larvae for Dpn (a marker of all central brain NBs), Mir (a marker of proliferating NBs), and Mod using a custom anti-Mod antibody (S1 Fig). Imaging of the entire CNS revealed ubiquitous Mod expression, including in cells throughout the central brain (CB), optic lobe (OL), and the ventral nerve cord (VNC), both in NBs as well as their differentiated progeny (Fig 4A–4D). Closer inspection found that Mod localized to distinct cellular compartments throughout different cell cycle stages. Mod was most prominently detected in a subnuclear compartment of interphase NBs overlapping with Fibrillarin (Fib), consistent with nucleolar localization (Fig 4E–4H and 4I–4L). Diffuse Mod signal could be seen in the nucleoplasm, along with faint signal in the cytoplasm of NBs (Figs 4H and S2). These signals were all impaired following allelic or RNAi-mediated Mod loss (S1 Fig). Previous studies have found similar patterns of Mod localization in other tissues, and nucleolin is known to undergo nucleocytoplasmic shuttling [52,56,60,77]. During mitosis, Mod was localized to the peri-chromosomal regions as well as throughout the cytoplasm, comparable to its localization in other mitotic tissues, and also similar to nucleolin in human cells along with other notable nucleolar proteins (Fig 4M–4P and 4X) [42,56,78]. Mod was also detected in Repo+ glia (Fig 4Q–4T) and ELAV+ neurons (Fig 4U–4X). We conclude that Mod is expressed throughout the larval CNS, including NBs.

The Mod localization patterns identified here and elsewhere provide a rationale for the RNA interactions uncovered in our RIP-seq analysis. The pronounced nucleolar Mod accumulation likely affords interaction with the identified rRNAs as well as snoRNAs, which play essential roles in rRNA modification and processing and may impact gene expression more broadly [79]. The less prominent, diffuse nuclear and cytoplasmic localization would likely represent opportunity for mRNA interactions and regulation. Dynamic changes during the cell cycle, which NBs undergo consistently throughout neurogenesis, may provide additional control of Mod-RNA interactions.

### Mod loss does not affect NB quiescence

**Fig 5:**
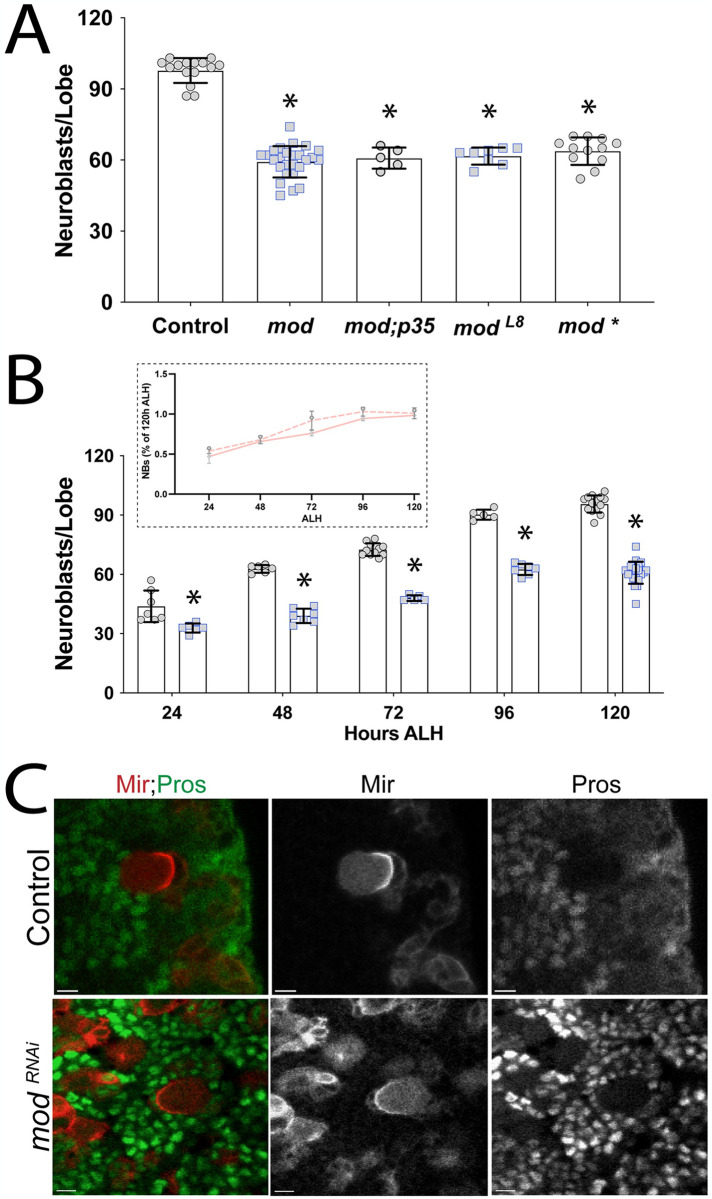
Mod loss is associated with a reduction of central brain NBs. **(A)** Graph depicting the number of Mir+ central brain NBs in Control (1407>*yw*), m*odRNAi* (*mod*), m*odRNAi* + p35 (*mod;p35*), the loss-of-function *mod* allele (m*odL8*), and an alternative m*odRNAi* line for confirmation (mod*) genotypes in L3 larvae. In both RNAi lines, Mod knockdown results in a significant reduction of the Mir+ NB pool, which is not rescued by overexpression of the anti-apoptotic p35 protein. Similar NB loss was measured in the *modL8* allele and alternate RNAi genotypes. **(B)** Developmental time course of central brain larval NB populations. Larva were dissected at 24, 48, 72, 96, and 120h ALH and the Mir+ NB number was assessed. Despite most NBs being present at 24 ALH, Mod-depleted brains consistently have fewer NBs compared to Control (1407>*yw*) across these developmental time points. However, the rate of NB expansion throughout larval neurogenesis is similar between control and Mod-depleted brain **(*inset*)**. Error bars represent the mean ± standard deviation; *, p *< 0*.*001* compared to Control; One-way ANOVA with Tukey’s multiple comparisons **(A)** or Student’s t-test with Bartlett’s correction **(B)**. **(C)** Representative images (from a total of 20 NBs examined from 20 larval brains each) of Control (1407>*yw*) and m*odRNAi* NBs showing that Pros is not detected in NBs nuclei.

The nucleolus houses essential processes including ribosome subunit biogenesis and rRNA transcription, both fundamental to cell growth and tissue development. Several nucleolar proteins have been described to play additional, non-ribosomal functions as well (e.g. genome stability, cell identity, and cell cycle regulation [80]). Localization of Mod to the nucleolus of interphase NBs prompted us to assess its role in their growth and proliferation. We expressed UAS-RNAi directed against Mod (*mod*RNAi) using the NB-specific 1407-GAL4 line and stained L3 brains with Dpn to mark all NBs [81]. Expression of *mod*RNAi reduced Mod protein levels in NBs compared to control (S1 Fig). Notably, Mod knockdown resulted in a modest but statistically significant decrease (~20%) in the number of Dpn+ central brain NBs, which are stereotypically numbered at ~100 in control brains (Fig 5A). Analysis of brains from the loss-of-function allele, *mod*L8, revealed NB loss similar to *mod*RNAi conditions. Larval NB proliferation begins after a 24-h quiescence following embryonic neurogenesis [82,83]. Exit from quiescence is mediated by mitogenic signals and nutrient sensing [3,84,85], and failure to reactivate from quiescence could explain the observed loss of NBs. To determine if NB exit from quiescence is perturbed and assess their abundance through larval development, we dissected larval CNS at 24, 48, 72, 96, and 120h ALH and quantified central brain NB number using Mir as a marker of proliferating NBs (Fig 5B). We found that *mod*RNAi brains had a NB number slightly lower than control brains 24h ALH, suggesting most NBs successfully reactivate at this early time point. Although fewer in total number compared to control at subsequent time points, the number of NBs increased in both control and *mod*RNAi brains across developmental time points, peaking at ~100 and ~65 NBs, respectively (Fig 5B). As a percentage of total NBs at 120h ALH, the rates of NB number increases were similar between control and *mod* RNAi conditions (Fig 5B inset). Although they do not entirely rule out a defect in reactivation, these results are not consistent with a significant contribution of quiescence reactivation at the embryonic-to-larval transition.

Inappropriate activation of quiescence can occur during later larval stages despite normal NB reactivation at larval neurogenesis onset. Numerous regulators including cell cycle proteins and chromatin modifiers are associated with induction of quiescence [86]. These proteins activate dormancy in response to stress, damage, or changes in nutrient availability. Although *mod*RNAi brains did not appear to present major defects in NB reactivation, it is possible that larval NBs become quiescent later in development. Quiescence can occur via low-to-moderate nuclear levels of the differentiation factor Pros. To test the possibility that loss of Mod initiated Pros-mediated quiescence, we assessed Pros localization in *mod*RNAi NBs. We found that NBs did not express nuclear Pros, suggesting these NBs are not dormant due to mislocalization and activity of Pros (Fig 5C). Together, these results indicate that *mod*RNAi NBs do not become quiescent due to Pros functions. Additionally, they suggest that NBs in Mod-depleted brains largely retain the ability to reactivate from quiescence and do not display evidence of subsequent quiescence induction.

### Mod loss impairs cell cycle progression

**Fig 6:**
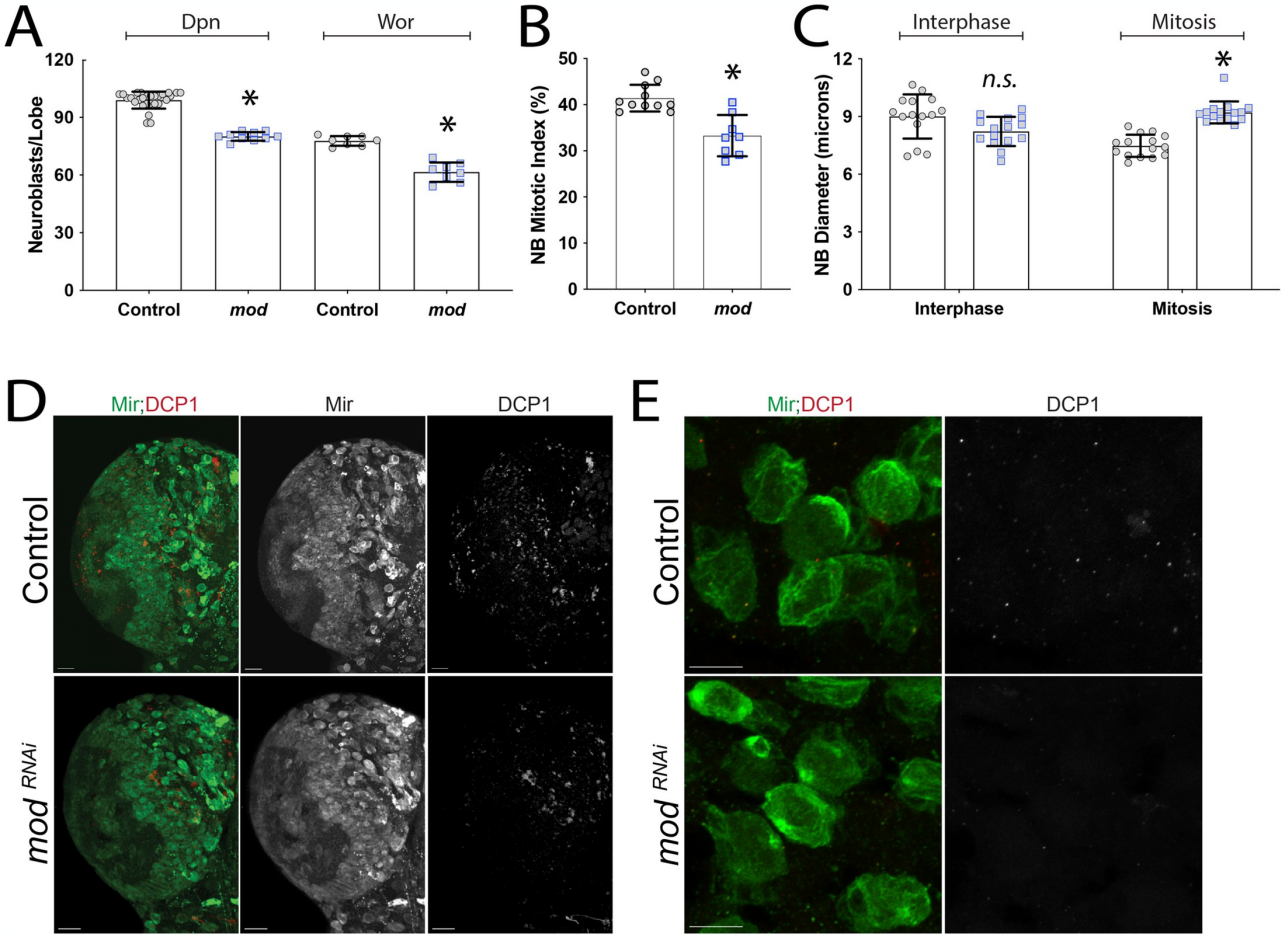
Loss of Mod causes cell cycle defects. **(A)** Deadpan (Dpn) and Worniu (Wor) positive NBs were quantified in Control (1407>*yw*) and *modRNAi* L3 central brain lobes, with *modRNAi* leading to a significant reduction in each. **(B)** Graph depicting the percentage of NBs in mitosis, marked as Mir+,PH3+. Mod knockdown significantly reduces the mitotic index of central brain NBs. **(C)** Graph showing the diameter of interphase and mitotic NBs in Control and *modRNAi* central brain lobes. Interphase NBs are of similar size (*n*.*s*., not significant), whereas mitotic *mod* NBs are enlarged compared to Control. NB diameters were measured from at least 3 brains. Error bars represent the mean ± standard deviation. *, *p < 0*.*01* compared to respective Control; One-way ANOVA with Tukey’s multiple comparisons **(A,C)**, Student’s t-test **(B)**. **(D)** Representative images for DCP1 showing Z-projections of individual lobes (Green: Mir, Red: DCP1). Few to no NBs were identified as DCP1+ in either Control or *modRNAi* conditions. Scale bars represent 20μm. **(E)** Higher magnification images, relative to (D), showing areas with NB clusters absent for DCP1 signal in both Control and *modRNAi* brains. Scale bars represent 10μm.

Although quiescence did not appear to account for the loss of NBs, we next assessed NB identity markers that could distinguish additional aspects of their proliferative status. Proliferative NBs express a unique combination of cell identity markers during larval neurogenesis, and loss of these markers is associated with aberrant cell cycle progression [87] and stem cell identity defects [88]. Specifically, proliferating NBs express Mir and Wor in addition to the ubiquitous NB marker Dpn [89,90]. We found that Mod knockdown caused a more substantial decrease in the number of Mir+ NBs compared to its reduction of the Dpn+ NB population (~80 Dpn^+^ vs. ~59 Mir^+^ NBs in *mod* RNAi brains; compare Figs 5A to 6A); that is, Mod knockdown appeared to have a stronger impact on the number of proliferative, Mir+ NBs. Stated otherwise, it can be inferred that a subset of ~20 of the Dpn+ NBs do not express Mir following Mod knockdown, consistent with these NBs having lost Mir expression without losing NB identity [89]. Next, we stained *mod*RNAi brains for Wor, a snail family zinc finger transcription factor that promotes NB proliferation and helps with maintenance of stem cell identity. We found that *mod*RNAi brains also had fewer Wor+ NBs compared to control brains (Fig 6A). Notably, the number of Wor+ NBs identified was similar to that measured for Mir+ NBs (Fig 5A), consistent with *mod*RNAi NBs having defects in cell cycle advancement and proliferation.

To assess the mitotic index of proliferating NBs, we co-stained brains with Mir, which is primarily cytoplasmic during interphase but forms a distinct polarized basal crescent during mitosis [91], and the mitotic chromosome marker PH3. We found that Mod knockdown caused a significant decrease in the number of PH3+ NBs compared to control, consistent with reduced mitotic index and a potential indicator of proliferation defects. Similarly, the percentage of NBs showing basal Mir crescents was also reduced following *mod*RNAi (Control: 18.6% vs. *mod*RNAi: 14.8%, p<0.01, Student’s t-test). Rapid divisions without overall cell growth occur during embryonic NB development, whereas larval NBs regrow after each division. Inappropriate NB size could stall cell division and lead to identity defects and cell cycle exit. To determine if Mod impacts NBs regrowth prior to mitosis, we measured the diameter of PH3^**+**^ and PH3- NBs in control and *mod*RNAi brains. NBs negative for PH3 were similar in size compared to control (Fig 6C), indicating that Mod knockdown does not prevent NBs from regrowing before entering mitosis. Interestingly, NBs positive for PH3 were found to be enlarged compared to control cells (Fig 6C). In previous studies, enlarged NBs accompanied by a decreased mitotic index have been associated with G2 arrest and mitotic errors [92]. In *Drosophila* sensory organs, these qualities decrease self-renewal capabilities and promote differentiation mechanisms [93].

Finally, to assess if NB proliferation defects lead to apoptosis, we overexpressed the anti-apoptotic protein p35 but found that this did not prevent loss of NBs in response to *mod*RNAi expression (Fig 5A). Additionally, death caspase-1 (DCP-1) staining did not show increased cell death in NBs, although we did observe DCP-1 signal in the tissue surrounding NBs (Fig 6D and 6E). These results together suggest that NBs are not lost to apoptosis. Overall, we conclude that Mod knockdown results in a non-apoptotic reduction in the neural stem cell pool that is associated with defects in cell cycle proliferation.

### Mod loss leads to abnormal expression of cell fate markers

**Fig 7:**
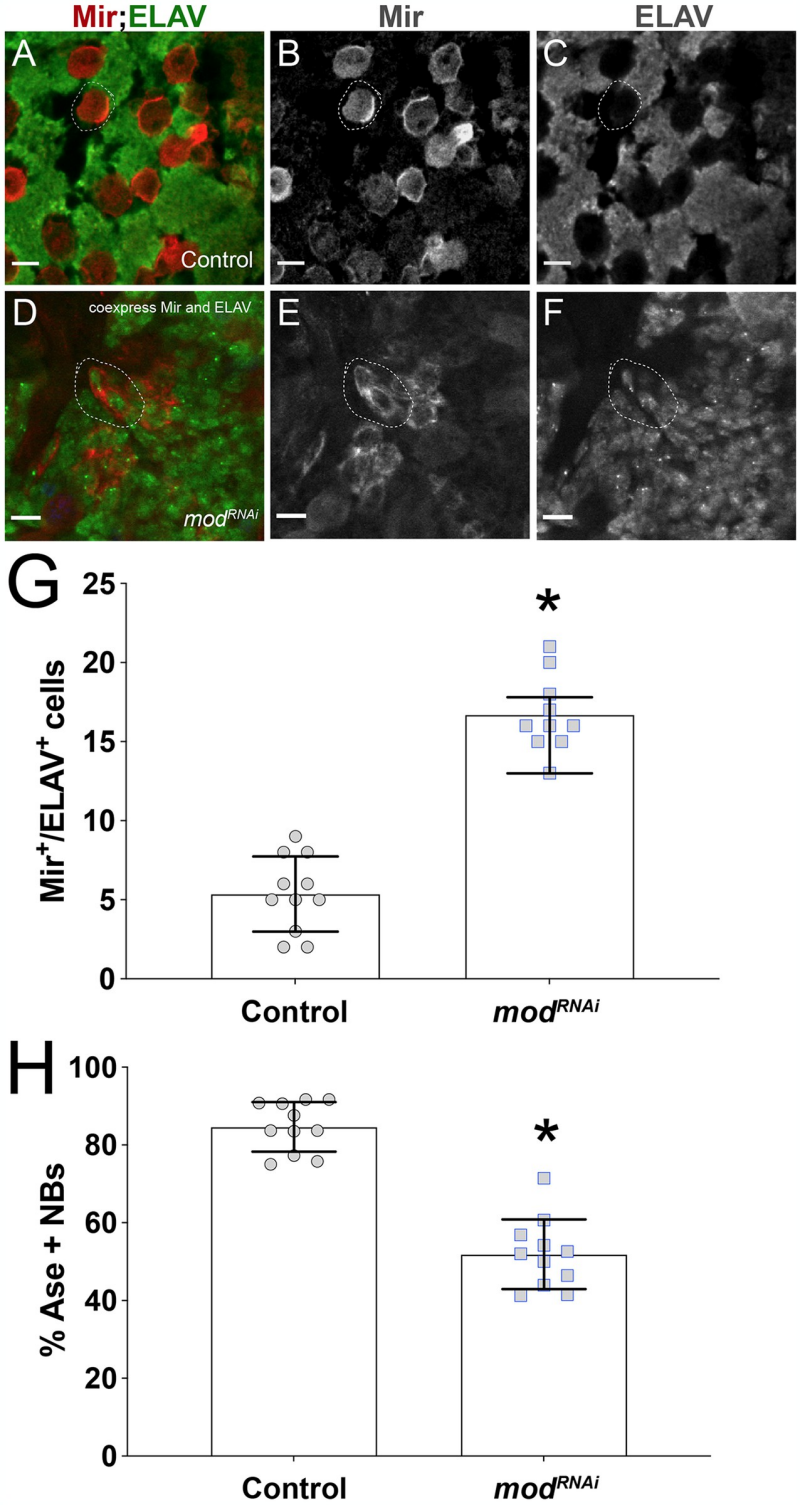
NBs in Mod-depleted brains display defects in expression of key cell identity factors. **(A)** Merged image depicting Control (1407>*yw*) brain marked with Mir+ NBs (red) adjacent to differentiated neural progeny, marked with ELAV (green) in a mutually exclusive manner. Individual channels are depicted in greyscale in panels **(B-C)**. **(D)** Merged image of cells in m*odRNAi* brain co-expressing Mir and ELAV. Individual channels are depicted in greyscale in panels **(E-F)**. Scale bars: 10μm. **(G)** Quantification showing numbers of Mir+/ELAV+-double positive cells in Control and m*odRNAi* brains. **(H)** Quantification showing percent of NBs expressing Ase in Control and m*odRNAi* brains. *p* < 0.0001 compared to Control; Student’s t-test with Welch’s Test. Error bars represent the mean ± standard deviation.

Next, we pursued other possible mechanisms for the observed NB loss, namely defects in cell identity markers that could trigger their premature differentiation. NBs normally express factors that aid in self-renewal and maintenance of stemness, and loss of these has been associated with premature differentiation [19]. Our RiP-Seq analysis identified the conserved neuronal differentiation factor, Embryonic lethal abnormal vision (ELAV), as a highly enriched Mod target (10th ranked FPKM value; S1 Table). Abnormal expression of ELAV in *mod*RNAi NBs could lead to their premature differentiation, thus we assessed ELAV expression in brains following Mod knockdown. Notably, we found a significant population of small Mir+ cells that co-express cytoplasmic ELAV, a phenotype that was rarely seen in control brains (Fig 7A–7G). This co-expression of Mir and ELAV effectively represents a mixed NB/GMC identity, consistent with gradual differentiation [94–96]. We conclude that the reduction in the number of NBs following Mod loss may be in part due to ELAV misexpression.

Mir, Dpn, and Wor are expressed in both Type I and Type II NBs. Each NB type also has lineage-specific factors [1]. Apart from Dpn, NBs also express a group of proneural genes called the *achaete-scute* gene complex (AS-C). Interestingly, all four members of the conserved AS-C gene cluster (e.g. achaete, scute, lethal of scute, and asense) were identified as top 10% ranked Mod targets (S1 Table). This group of bHLH transcription factors plays an essential role in specifying sense organ formation in the peripheral nervous system as well as in initiating NB fate in the central brain [97,98]. Thus, defective expression of AS-C components could be responsible for NB loss. Ase is part of a core set of transcription factors involved in NB self-renewal and is restricted to the Type I lineage [99], thus we analyzed Ase expression following *mod*RNAi by co-staining brains for Mir and Ase and quantifying the number of Ase+ NBs. Notably, Mod knockdown resulted in a decrease in the number of NBs expressing Ase (Fig 7H). Further studies will be required to decipher how Mod might regulate the function of Ase and other proneural targets, but these results illuminate a hitherto unidentified link with diverse genes essential to neurogenesis.

### Mod loss does not impair ACD

**Fig 8:**
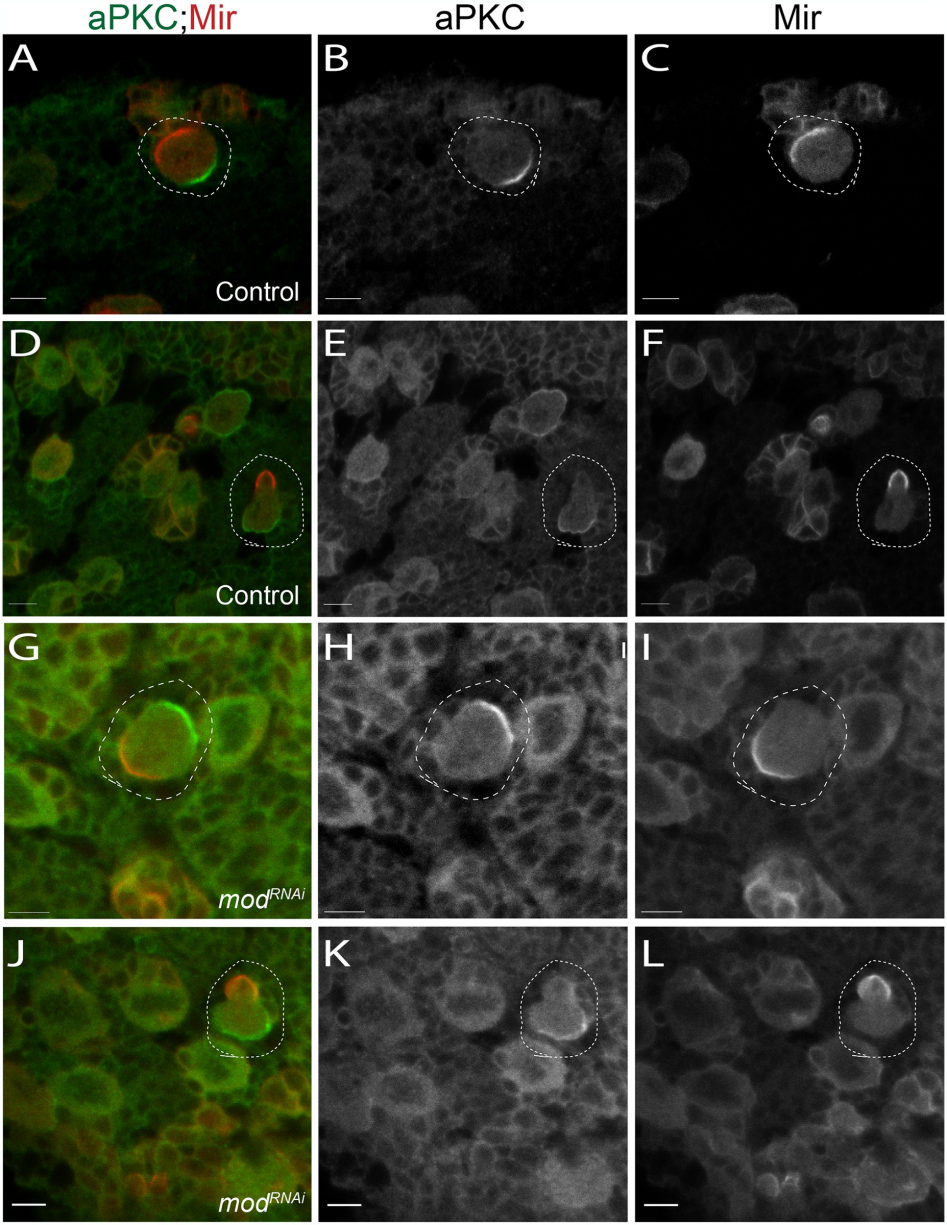
Loss of Mod does not affect NB asymmetric cell division. Images of Control (1407>*yw*) and *mod*RNAi-expressing *(mod*) NBs. **(A-C)** Control NBs showing basal localization of Mir (red) and apical localization of aPKC (green). These factors are asymmetrically segregated during telophase **(D-F)**. **(G-I)** *mod*RNAi NBs do not exhibit defects in aPKC or Mir polarity, nor do they show improper segregation of these factors during telophase **(J-L)**. Images are representative of at least 20 cells per genotype assessed. Scale Bars: 5μm.

During larval neurogenesis, NBs divide asymmetrically to self-renew while also producing differentiated cell types that will form the complex adult CNS. To achieve this, NBs establish apical-basal polarity and divide along this axis to produce unequally sized progeny (Fig 1C). Defects in ACD are known to disrupt NB homeostasis, either by expanding the stem cell pool or, conversely, resulting in premature NB differentiation [6,10,100]. We assessed these properties in *mod*RNAi-expressing NBs and found that NBs did not exhibit defects in apical-basal polarity, using apical aPKC and basal Mir as markers (Fig 8). Furthermore, telophase cells in both control and *mod*RNAi brains presented with a larger apical and smaller basal domain typical of normal size asymmetry (Fig 8D–8F and 8J–8L). We conclude that Mod knockdown does not impact ACD, ruling this out as a likely mechanism of the observed NB loss.

### Mod loss causes altered nucleolar architecture

**Fig 9:**
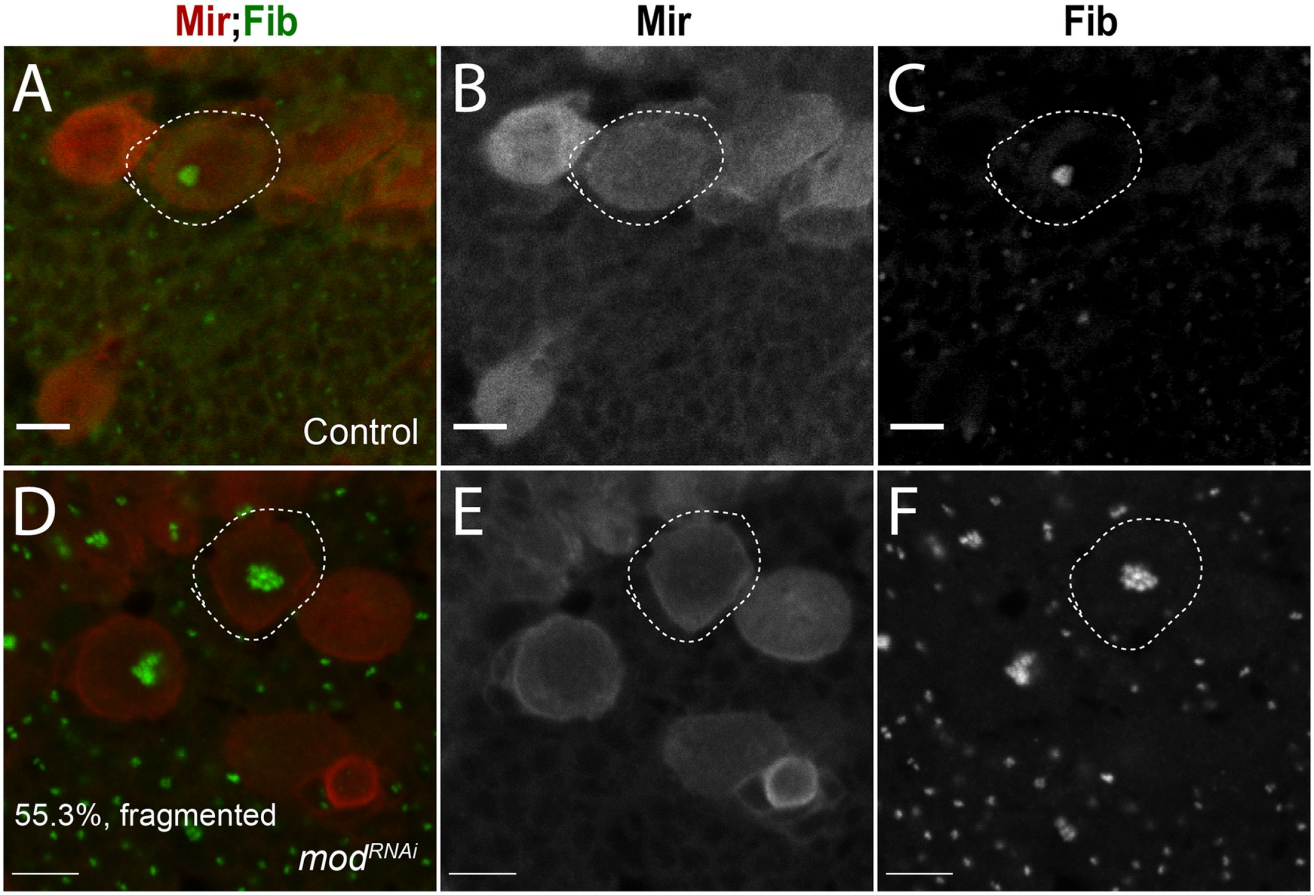
Mod is required to maintain nucleolar architecture. ** (A)** Merged and **(B-C)** individual greyscale panel images of Control NBs (1407>*yw*) stained with Mir (red) and Fib (green). Nearly all NBs have a single, concentrated fibrillarin-positive nucleolar region, indicative of normal nucleolar structure (n = 87 NBs). **(D)** Merged and **(E-F)** individual greyscale panel images of *modRNAi* NBs showing the predominant phenotype that presents as a cluster of ‘Fragmented’ fibrillarin-positive nucleolar foci (n = 85 NBs). Scale Bars: 5μm **(A-C)**, 7μm **(D-F)**.

Prior to mitosis, cells inactivate rRNA transcription and the nucleolus is disassembled. Proteins that are sequestered in the nucleolus are released and proceed to control cell cycle progression, DNA repair, and stress responses [101]. The nucleolar domain is reformed after mitosis from nascent and pre-existing components inherited from previous divisions [102,103]. The intact structure of the nucleolus depends on transcription of rRNA [104,105]. Functional ribosomes are assembled from precursor subunits that are produced from rRNA in the nucleolus, thus availability of these subunits ultimately promotes formation of the nucleolus. As such, the size and architecture of the nucleolus is indicative of the levels of rRNA transcription. This, along with observed downregulation of several genes involved in nucleolar assembly and rRNA processing obtained from our DGE analyses (Fig 3 and S2 Table), prompted us to examine nucleolar structure in *mod*RNAi NBs. Using Fib as a marker, we found that 55.3% of *mod*RNAi NBs contain clusters of fragmented nucleoli (Fig 9D–9F). In contrast, control NBs nearly always presented with a single, concentrated Fib focus (Fig 9A–9C). Thus, Mod knockdown appears to disrupt normal nucleolar structure in NBs, which is consistent with downregulation of rRNA processing genes and with phenotypes previously described following mutations in other *Su(var)* genes [106]. Furthermore, Mod loss could inhibit re-assembly of the nucleolus after mitosis and lead to the fragmented phenotype observed here, thus contributing to the neural stem cell loss [104]. Other studies have found strong links between nucleolar integrity and stem cell maintenance [107]. For instance, the nucleolar protein nucleostemin is required to maintain stem cell and cancer cell growth, with defects inducing cell cycle arrest and apoptosis [108]. Loss of another nucleolar protein Nopp140, which also participates in ribosome assembly, leads to nucleolar deformities and loss of stem cells similar to Mod [109]. Interestingly, unlike Mod, Nopp140 depletion triggers NB apoptosis, suggesting neural stem cells respond differently to dysfunction of unique nucleolar genes. While loss of Mod causes a reduction in the pool of central brain NBs, a population of stem cells remain, although a considerable fraction of them present a molecular signature consistent with errors in proliferation [109,110]. Although knockdown of Mod leads to loss of NBs via an apoptosis-independent mechanism, these studies collectively highlight a critical link between nucleolar integrity and maintenance of neural stem cell homeostasis.

Previous studies have described the importance of Mod throughout development. To our knowledge, none have provided a comprehensive analysis of its role in specific developmental stages. We have described the function of Mod in neural stem cells during the essential phase of larval neurogenesis. Overall, our results identify Mod as a regulator of stem cell homeostasis and suggest a potentially complex mechanism involving predicted roles in nucleolar maintenance and rRNA processing as well as the unexpected link to key neural identity and neurogenesis-promoting genes.

## Conclusion

Although early studies provided enlightening details about the structure and function of Mod, a more comprehensive understanding of its molecular targets has been a notable knowledge gap. Our transcriptome-wide RiP-Seq target analysis combined with differential gene expression studies provides more information about these targets, (1) confirming predicted targets involved in ribosome function and cell growth, and (2) suggesting unanticipated, novel roles in neurogenesis and stem cell identity. We found that loss of Mod activates a cellular response involving downregulation of rRNA processing in the nucleolus and cytosol (GO: 0006364). These changes are accompanied by downregulation of multiple components of cell proliferation including genes involved in DNA replication (GO: 0006261) and prometaphase (GO: 0000236) as well as the familiar epidermal growth factor receptor (Egfr). Separately, nervous system regulatory genes were also downregulated. The most significant targets included Notch, and various Notch signaling components such as bib and the transcriptional regulator grainy head (grh; Fig 3).

*In vivo* studies further solidified the important role of Mod in neurogenesis and cell fate determination, as suggested by our transcriptomic and target analyses. Enrichment of neurogenesis targets and the categories of differentially expressed genes encouraged us to further investigate Mod in the fruit fly brain. Knockdown of Mod resulted in cell cycle progression defects and loss of key stem cell identity factor expression in NBs. These cells also exhibited growth defects and cell cycle errors previously associated with stem cell senescence [111]. While the specific details underlying the arrest remain unknown, some possibilities can be inferred from the DE and target analyses. Disruption of rRNA processing is one potential source of cell cycle arrest [112]. Furthermore, the fragmented nature of the nucleolus implies deficiencies in nucleolar dynamics, which may be attributed to some of the ribosomal protein targets identified in our analysis (Fig 2E). Of the differentially expressed genes, downregulation of Egfr, bib, fng, and disabled (Dab) point to numerous abnormalities in nervous system development and cell specification pathways. A closer look at the cell population impacted revealed that the Type I lineage is highly sensitive to Mod loss. The basic-helix-loop-helix protein Ase is restricted to Type I NBs and its signaling occurs in conjunction with Delta-Notch mechanisms during early development [113]. Thus, maintenance of the Type I lineage throughout larval development may require Notch and Ase functions. Ase expression is also required for the transition of neuroepithelial cells to neuroblasts in the optic lobe [99,113].

Our studies provide new details about the molecular functions of Mod in proliferative stem cells, notably in NBs. Although our target analysis was performed in S2 cells, it is likely that some fundamental mechanisms and interactions are conserved *in vivo*. Similar analyses performed in S2 cells demonstrated the interaction between Notch receptor and its ligand, Delta, along with numerous other interactions that have been confirmed *in vivo* [114–116]. Future studies will be required to determine the precise mechanisms for how Mod regulates target RNAs and controls gene expression during neural development.

## Supporting information

S1 Fig Mod antibody and RNAi validations. **(A)** Merged image depicting Mod protein localization using anti-Mod antibody (blue), together with the nucleolar marker Fibrillarin (Fib; green) and NB marker Miranda (Mir; red), in a Control NB (1407>*yw*). Individual channels are depicted in greyscale in **(B-D)**. **(E)** Merged image of staining of *modL8* NBs using the same antibodies and imaging settings as above with individual channels represented in **(F-H)**. NBs in *modL8* brains are negative for Mod signal and total signal is abrogated. Scale bars represent 5μm. **(I-P)** Larval brains immunostained with Mir (Red), Fib (Green), and Mod (Blue) in Control **(I-L)** or *modRNAi* **(M-P)** using identical antibodies and imaging settings. RNAi expression decreases Mod signal in NBs. Scale bars represent 10μm.
(TIFF)

S2 Fig Mod localizes both within and outside the nucleolus in NBs. **(A)** Merged image depicting Mod protein localization (green), together with the nucleolar marker Fib (red) and NB marker Mir (blue), Individual channels are depicted in greyscale in panels **(B-D)**. In panel **(C)**, white arrows indicate strong nucleolar Mod signal, whereas yellow arrows indicate diffuse straining outside the nucleolus in non-mitotic cells. Red arrow indicates diffuse Mod localization in a mitotic NB. **(E-H)** Higher magnification representation and instead using Lam antibody to mark the nuclear envelope (red). In panel **(G)**, white dashed line outlines the nucleus, whereas the yellow dashed line marks the NB periphery. Similar to **(C)**, strong Mod signal is found in a discrete subnuclear structure consistent with the nucleolus, whereas fainter and diffuse signal is found in the nucleoplasm and cytoplasm. Scale bars represent 10μm.
(TIFF)

S1 Table RNA targets of Mod identified by RiP-Seq. (XLSX)

S2 Table Differentially expressed genes following Mod knockdown. (XLSX)
