# Supplementary figures and images for: The RNA-binding protein Modulo promotes neural stem cell maintenance in Drosophila
Source: PLoS One. 2024 Dec 19;19(12):e0309221. doi: 10.1371/journal.pone.0309221 (PMC11658480; doi:10.1371/journal.pone.0309221)

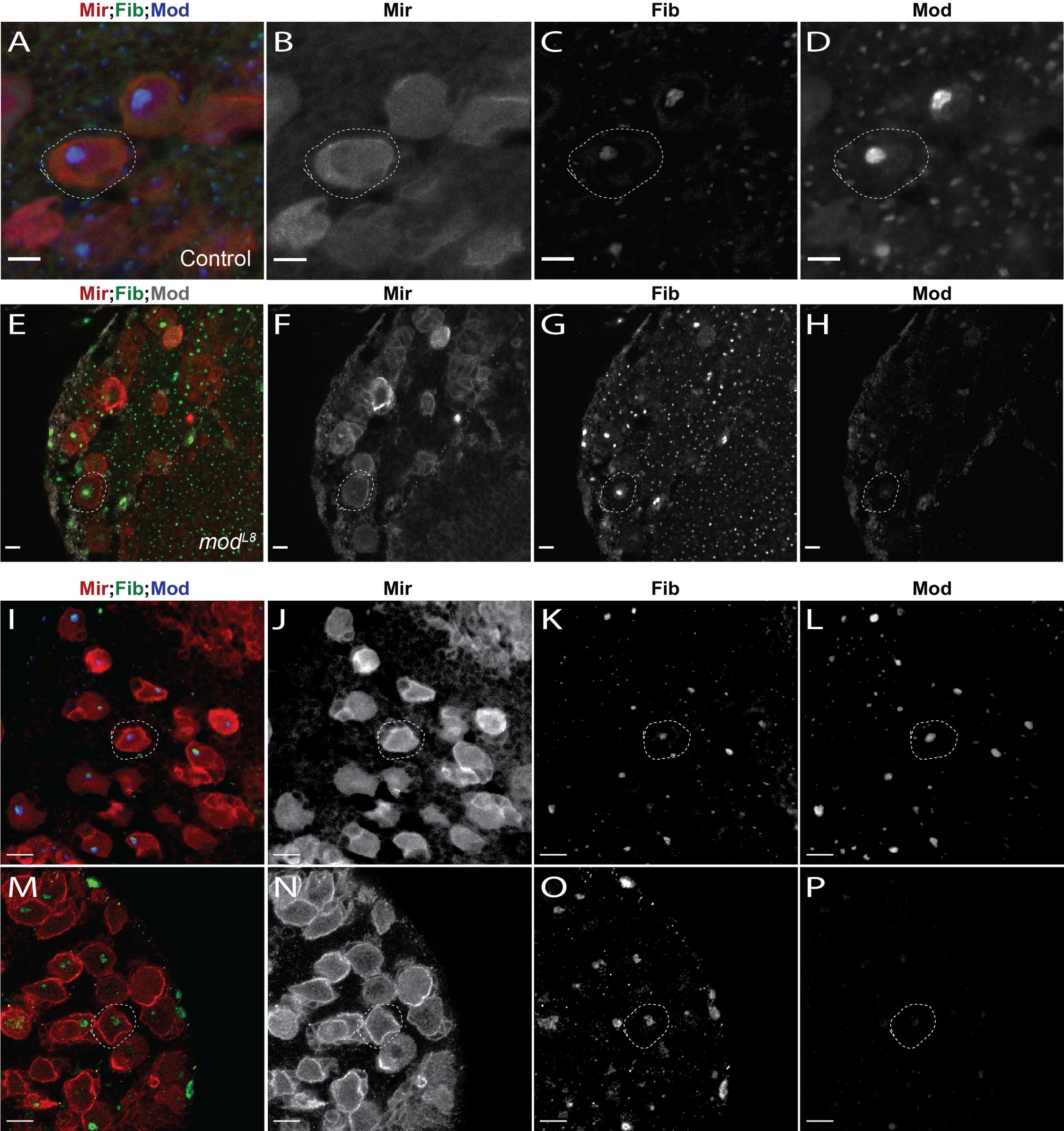

Supplement: S1 Fig — (A) Merged image depicting Mod protein localization using anti-Mod antibody (blue), together with the nucleolar marker Fibrillarin (Fib; green) and NB marker Miranda (Mir; red), in a Control NB (1407>yw). Individual channels are depicted in greyscale in (B-D). (E) Merged image of staining of modL8 NBs using the same antibodies and imaging settings as above with individual channels represented in (F-H). NBs in modL8 brains are negative for Mod signal and total signal is abrogated. Scale bars represent 5μm. (I-P) Larval brains immunostained with Mir (Red), Fib (Green), and Mod (Blue) in Control (I-L) or modRNAi (M-P) using identical antibodies and imaging settings. RNAi expression decreases Mod signal in NBs. Scale bars represent 10μm. (TIFF) [file pone.0309221.s001.tiff]

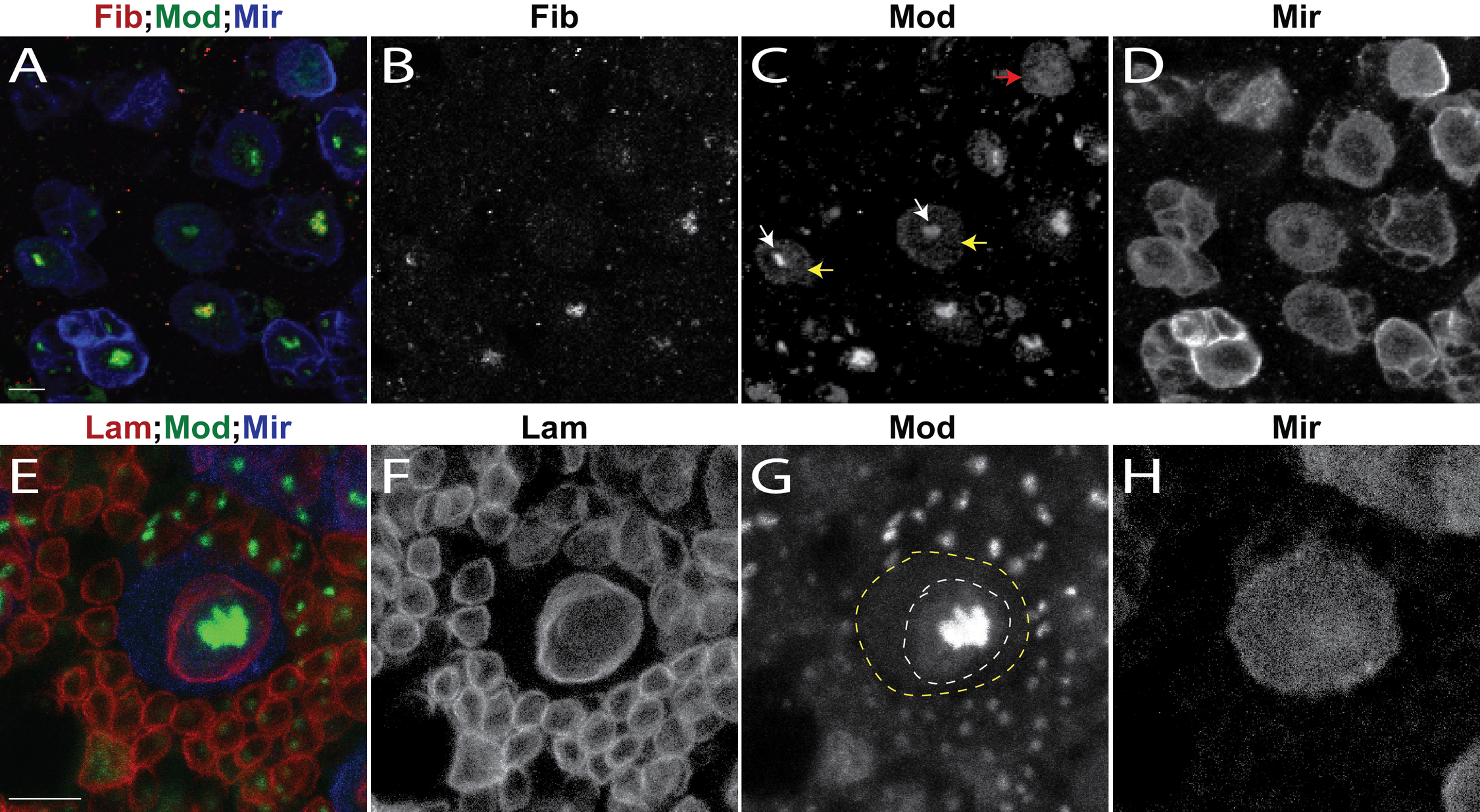

Supplement: S2 Fig — (A) Merged image depicting Mod protein localization (green), together with the nucleolar marker Fib (red) and NB marker Mir (blue), Individual channels are depicted in greyscale in panels (B-D). In panel (C), white arrows indicate strong nucleolar Mod signal, whereas yellow arrows indicate diffuse straining outside the nucleolus in non-mitotic cells. Red arrow indicates diffuse Mod localization in a mitotic NB. (E-H) Higher magnification representation and instead using Lam antibody to mark the nuclear envelope (red). In panel (G), white dashed line outlines the nucleus, whereas the yellow dashed line marks the NB periphery. Similar to (C), strong Mod signal is found in a discrete subnuclear structure consistent with the nucleolus, whereas fainter and diffuse signal is found in the nucleoplasm and cytoplasm. Scale bars represent 10μm. (TIFF) [file pone.0309221.s002.tiff]
